# Supplementary material for: Pre-warming before general anesthesia with isoflurane delays the onset of hypothermia in rats
Source: PLoS One. 2020 Mar 3;15(3):e0219722. doi: 10.1371/journal.pone.0219722 (PMC7053737; doi:10.1371/journal.pone.0219722)
Supplement: Attachment — Submitted filename: 2_author response_Pang_PLoS_appeal_PONE-D-19-18070.rtf [file pone.0219722.s001.rtf]

>>Thank you to the Academic Editor and reviewers for their time in reviewing our manuscript. We respectfully disagree with the Acad Editor's reject decision and hope to have justified this in our response to each comment, below. Our comments are indicated by ?
">>" with changes to the manuscript highlighted.

PONE-D-19-18070
Pre-warming before general anesthesia with isoflurane delays the onset of hypothermia in rats
PLOS ONE

Dear Dr. Pang,

Thank you for submitting your manuscript to PLOS ONE. After careful consideration, we have decided that your manuscript does not meet our criteria for publication and must therefore be rejected.

>>We have submitted an appeal to this decision as we do not feel that it is justified based on the comments of the Acad Editor and those of the reviewers. In addition to the responses provided here, an overview of our responses to the specific comments of the Acad Editor are provided in a cover letter.

Specifically:
 
The authors investigated theeffect of pre-warming at delaying the onset of hypothermia during general anesthesia. They compared different pre-warming temperature regimens and performed a comprehensive analysis. However, there were enormous concerns regarding the current manuscript.
1. Reviewers and I all especially concerned about the novelty of the article.

>>We are greatly surprised that novelty has been used, and appears to be the determining factor, in justifying the decision for rejection.
On a fundamental level, it is well-established and has been well-publicised that PLOS One does not use novelty as a criterion for acceptance. This is clearly stated on several pages of the journal's website: 
"We evaluate submitted manuscripts on the basis of methodological rigor and high ethical standards, regardless of perceived novelty."
(https://journals.plos.org/plosone/s/journal-information).
"The editors make decisions on submissions based on scientific rigor, regardless of novelty."
(https://journals.plos.org/plosone/s/editorial-and-peer-review-process)

Based on this, it would not seem appropriate to base the decision to reject on a claimed lack of novelty. Nevertheless, we are happy to address this criticism of the Acad Editor as we feel it reveals a bias underlying the reject decision that our work is intended to improve human healthcare. As the Acad Editor is aware, pre-warming has been rigorously studied in humans and is widely-accepted as beneficial in limiting peri-anaesthetic hypothermia. This is not the case in animal species, where there are few (n = 3) published studies, the conclusions from which are far from clear. Our work focuses on the health and welfare of animal species, not humans. There was never an intention that our study served to promote rats as a model for peri-anaesthetic hypothermia in humans. We apologise if this was unclear or open to interpretation, but we feel that there were numerous indicators of our intention:
1. This is reflected in the Introduction, where the key veterinary literature on hypothermia is cited. The human literature is cited where necessary to complement what is known/unknown in veterinary species. 
2. One of the study objectives was to evaluate the use of proxy sites for core temperature estimation (tail and fur); these are specific to veterinary species. 
3. Approximately half of the Discussion is focused on the incidence and implications of hypothermia in veterinary species.
4. The focus on veterinary species was described in the covering letter accompanying our submission ("Pre-warming has been successfully applied in human medicine and is now a standard of care. In contrast, it has received little investigation in animal species.").

The available veterinary literature on pre-warming before general anaesthesia is sparse. To our knowledge, there are 3 published studies on pre-warming in veterinary medicine (based on a PubMed search using term pre-warming AND hypothermia AND (dog OR cat OR rat OR mouse OR veterinary) and search of reference lists). One of these studies is our previous work in rats (PMID: 28599579) and the other two are in dogs (PMIDs: 28216687, 26383752). Our first paper on this subject was a proof of concept study, which showed the potential for applying pre-warming in rats. This follow-up study used the results of the initial work as a basis for quantifying the efficacy of different pre-warming strategies. This is the only study to quantify pre-warming in a veterinary species. Our goal was never to use rats as a model for peri-anaesthetic temperature management in humans.
 
2. The effectiveness and clinical implicationof pre-warming treatment could not be ensured.Specially, the protection of pre-warming against hypothermia was no more valuable, because in clinical practice, the duration of anesthesia is often longer than 15 minutes or even hours. In addition, the core temperature is greatly affected by numerous environmental factors, not just general anesthetics.

>>It was never our intention to imply that this work was for the ultimate benefit of humans i.e. using rats as a model for studying peri-anaesthetic hypothermia in humans. The phrases "clinical implication" and "clinical practice" suggest that our work is being viewed in the context of human anaesthetic procedures. Were this the case, we would agree with the Acad Editor that our results do not support pre-warming as a useful strategy in humans. Pre-warming has already been well-studied, and its efficacy established, in humans (see work of DI Sessler). Importantly, humans themselves are the most appropriate and valid clinical model to study this subject; there is no need to use an animal model. Interestingly, in performing our study we have shown that the assumption of back translation (from humans to rats) is flawed - we did not draw attention to this in the paper, again, because any relationship to humans was unintended. 

Related to our comment that humans themselves are the appropriate model to to study this subject, the assumption that rats could serve as models of hypothermia in humans is flawed (fundamental differences in body mass, body surface area, kinetics of heat distribution). On this basis it would not make sense for us to have chosen rats to study peri-anaesthetic hypothermia in humans. 

The interpretation of our results, that pre-warming is effective but has a limited duration of effect, is strictly in the context of the frequently short (< 30 minutes) anaesthetic procedures performed in rats (castration, ovariohysterectomy, mass removal…). Furthermore, it is clearly stated that "longer duration procedures would require additional heating support" (Abstract) and "The duration of effect was short, necessitating temperature support for longer anesthetic periods" (Conclusion). At no time did we claim to have found a solution to avoid hypothermia for procedures lasting hours.

We do not contest that core body temperature is influenced by multiple factors; however, as numerous publications in humans show (supported by our telemetric data) the single most important determinant of the initial drop in body temperature following induction of general anaesthesia is adverse effect of general anaesthetics on thermoregulation (key references were cited in the paper - please see Matsukawa, Sessler et al. 1995, Matsukawa, Kurz et al. 1995, Xiong et al. 1996, Kurz et al. 1995, Torossian 2008). The role of environmental factors is secondary during this initial anaesthesia-induced reduction in temperature.

3. The safety of pre-warming treatment was another important issue. The upper limit of 40°C was selected based on the results that the core temperatures in mice of 41.5 ± 0.1°C for 2 hours could resulted in apoptosis. However, it was not yet proven whether 40°C has a long-term effect on brain function. It is essential to assess intraoperative and postoperative neurobehavioral outcomes.
Unfortunately, the currentmanuscript could not give the readers more valuable clinical significance and evidence-based conclusions. 

>>We do not contest the Acad Editor's assertion that the long-term effects of a 40°C core body temperature on brain function are unknown. The 40°C treatment group was selected to provide a comparison with the 1% increase in core temperature group (the temperature increase found to be successful in humans). We cannot comment on the long-term effects of warming to 40°C as this was not a goal of this study. Evaluation of behaviours indicative of stress suggest that there was similar stress in the 40°C and 1% groups (see Behavioural assessment section). Given that our data (Fig. 1) show a limited advantage of warming to 40°C versus a 1% core temperature increase we did not recommend warming to 40°C. The Limitation section has been revised to underline this point: "Any adverse effects of the transient increase in core temperature to 40°C are unknown as neurological outcomes were not studied following recovery; however, no overt changes in behavior or body condition were noted in the weeks following the study."

I am sorry that we cannot be more positive on this occasion, but hope that you appreciate the reasons for this decision.

Yours sincerely,

JianJun Yang, M.D., Ph.D.
Academic Editor
PLOS ONE


[Note: HTML markup is below. Please do not edit.]

Reviewers' comments:

Reviewer's Responses to Questions

Comments to the Author

1. Is the manuscript technically sound, and do the data support the conclusions?

The manuscript must describe a technically sound piece of scientific research with data that supports the conclusions. Experiments must have been conducted rigorously, with appropriate controls, replication, and sample sizes. The conclusions must be drawn appropriately based on the data presented. 

Reviewer #1: Yes

Reviewer #2: Partly
 
 


2. Has the statistical analysis been performed appropriately and rigorously? 

Reviewer #1: Yes

Reviewer #2: No
 
 
 
 


3. Have the authors made all data underlying the findings in their manuscript fully available?

The PLOS Data policy requires authors to make all data underlying the findings described in their manuscript fully available without restriction, with rare exception (please refer to the Data Availability Statement in the manuscript PDF file). The data should be provided as part of the manuscript or its supporting information, or deposited to a public repository. For example, in addition to summary statistics, the data points behind means, medians and variance measures should be available. If there are restrictions on publicly sharing data—e.g. participant privacy or use of data from a third party—those must be specified.

Reviewer #1: Yes

Reviewer #2: Yes
 
 
 
 


4. Is the manuscript presented in an intelligible fashion and written in standard English?

PLOS ONE does not copyedit accepted manuscripts, so the language in submitted articles must be clear, correct, and unambiguous. Any typographical or grammatical errors should be corrected at revision, so please note any specific errors here.

Reviewer #1: Yes

Reviewer #2: Yes
 
 
 
 


5. Review Comments to the Author

Please use the space provided to explain your answers to the questions above. You may also include additional comments for the author, including concerns about dual publication, research ethics, or publication ethics. (Please upload your review as an attachment if it exceeds 20,000 characters)

Reviewer #1: The authors aimed to investigate the effect of prewarming on the isoflurane-induced hypothermia in the rat. It was demonstrated that prewarming confers a protective effect against hypothermia during volatile anesthesia. The animal experiment seems to have been adequately performed. However, it is not clear what is the novel finding of this study.

>>Thank you for taking the time to review our manuscript. Regarding the criticism of a lack of novelty, we would refer the reviewer to the comments above, made in response to a similar concern from the Acad Editor. Briefly: 1. investigation of pre-warming in animals is scarce and, as our results show, findings from studies in humans do not translate to rats. 2. Novelty is not a criterion used by PLOS One in decisions regarding acceptance of papers. 3. This study was performed in the hope of improving per-anaesthetic care in rats. There was never an intention to translate our findings to humans.

1. Figures 1 and 2 seem to show the same data. How was the data in Figure 2 obtained? The authors should explain why Figure 2, in addition to Figure 1, is necessary in this paper.
>>The reviewer is correct, in that the data of Fig. 2 are derived from Fig 1. We elected to split the information in to 2 figures as we felt that this showed more clearly the key points we were hoping to make: 1. the overall temperature profile for the experiment and 2. the focus on the AUC analysis. In writing the paper we did try combining this information on to a single graph but felt that the end result was harder to interpret. In response to the Reviewer's comment we have combined the information in to a single figure (Fig 1).

2. Figure 3 is obtained from Figure 1. It is not clear why Figure 3 is necessary in this paper.
>>The data presented in Fig 3 differ from Fig 1 in that the times to hypothermia shown in Fig 3 are based on calculating an individual hypothermia threshold for each rat (as described in the Methods, line 152). This differs from the threshold shown in Fig 1, which is derived from the population data (as described in Methods, line 155). In revising Fig 1 (in response to previous comment), the broken vertical lines extended from the hypothermia threshold to the x-axis have been removed. In doing this, to keep the figure clear in the process of combining Figs 1 and 2, we believe that the clearest way to present the times to hypothermia is by keeping the bar graph of Fig 3 (now Fig 2).

3. Effectiveness of prewarming to maintain core temperature during anesthesia has been already reported (lines 371-376). The present study shows the effect of prewarming in more detail, but novelty compared with the previous report is not clear. The author should more clearly explain novelty of this research.
>>The paper cited is our previous work on this subject. In this previous study we showed that pre-warming was feasible but, critically, did not establish the potential duration of effect of pre-warming, allowing us to evaluate if pre-warming alone might be sufficient in preventing hypothermia for short procedures. The study reported here builds upon this previous work. We believe that the Reviewer's focus on novelty is misguided; we are seeking to build on previous work to better understand the strengths and limitations of pre-warming in rats.

4. Differences among core, rectal and fur temperature are as expected (Figures 4-6). What is the novel finding of this data?
>>We would respectfully refer the Reviewer to the editorial policy of PLOS One regarding novelty. Additionally, there have been claims in the literature that temperature measurements at other sites (e.g. fur) may serve as a suitable proxy for core temperature. We apologize if this was not clear in the manuscript and have added the following in the Discussion: "Comparing these alternative sites for temperature measurement with telemetric core temperature allows an accurate determination of their inter-relationship. These findings show that rectal temperature can be used as a proxy for core temperature in healthy rats but fur and tail temperatures are highly variable and unlikely to be useful in estimating core body temperature. The application of the Bland-Altman method reveals this variability, in contrast to interpreting correlation alone. [48]"

5. It is not clear why the behavioral analyses were necessary and what was suggested by the findings of the behavioral analyses.
>>The intention of the behavioral analyses was described in the Methods (line 162 - "for signs of behaviors associated with distress") with 2 references supporting our choice of behaviors (refs 21 and 22). The behavioral results are open to interpretation as, while the rats in the pre-warming groups showed a clear preference to face away from the heat source, this was not couple with more specific signs of distress. This was described in the Discussion (line 408).

Reviewer #2: PONE-D-19-18070
In this preclinical study, the Authors evaluated the prewarming animals before induction of general anesthesia would delay the onset of hypothermia. A secondary objective was to compare the accuracy of different temperature measurement sites to core temperature (telemetric capsules implanted in the abdomen).
This prospective, crossover study (n = 17 adult male and female SD rats) compared three treatment groups: PW1% (pre-warming to increase core temperature 1% over baseline), PW40 (pre-warming to increase core temperature to 40°C) and NW (no warming).
The PW1% group was completed first to ensure tolerance of pre-warming.
Treatment order was then randomized and alternated after a washout period. Once target temperature was achieved, anesthesia was induced and maintained with isoflurane in oxygen without further external temperature support.
Pre-warming was effective at delaying the onset of hypothermia, with a significant difference between PW1% (11.2 minutes) and PW40 (14.7 minutes, p = 0.0044 (95%CI -12 to -2.2), PW40 and NW (6.0 minutes, p = 0.003 (95%CI 1.8 to 8.7) and PW1% and PW40 (p = 0.004, 95%CI -12 to -2.2).
The rate of heat loss in the pre-warmed groups exceed that of the NW group: PW1% versus NW (p = 0.005, 95%CI 0.004 to 0.027), PW40 versus NW (p < 0.0001, 95%CI 0.014 to 0.036) and PW1% versus PW40 (p = 0.07, 95%CI -0.021 to 0.00066).
The Authors concluded that, pre-warming alone confers a protective effect against hypothermia during volatile anesthesia; however, longer duration procedures would require additional heating support.

Comments
The study confirms in various animal subset an information already proven in humons.
>>This is not the case. Pre-warming in humans is more successful than what we have been able to achieve here in rats. As previously presented in response to the Acad Editor, not all research is performed for the benefit of humans. This work, as we hoped had been clear from the Introduction and Discussion, particularly with the use of veterinary references, was intended to improve veterinary care of rats.


Not having a sample size calculation, it is not clear the strength of reported results and derived conclusions
>>The sample size estimation was presented in the Methods (line 107).

I would suggest to summarize the overall meaning of reported results in the first sentence of the Discussion section.
>>The main findings of the study were described in the opening paragraph of the Discussion - please see sentence beginning, "The main findings of this study…".

Discussion should be shortened (by 10 to 15% or more) and focused on clinical implication of the present study.
>>We have no objection to shortening the Discussion but would require specific directions from the Reviewer in identifying what they feel should be removed. As described above in our response to the Acad Editor, this study was never intended to be used for the "clinical" benefit of humans.

Please edit the Reference section according the journal requirements
>>Thank you for drawing our attention to these errors. We had downloaded the journal style format in to our referencing software and failed to notice the errors that had crept in. These have been corrected.
 
 
 
 


6. PLOS authors have the option to publish the peer review history of their article (what does this mean?). If published, this will include your full peer review and any attached files.


If you choose "no", your identity will remain anonymous but your review may still be made public.


Do you want your identity to be public for this peer review? For information about this choice, including consent withdrawal, please see our Privacy Policy.

Reviewer #1: No

Reviewer #2: No


[NOTE: If reviewer comments were submitted as an attachment file, they will be attached to this email and accessible via the submission site. Please log into your account, locate the manuscript record, and check for the action link "View Attachments". If this link does not appear, there are no attachment files to be viewed.]

- - - - -
For journal use only: PONEDEC3
 
 
 

In compliance with data protection regulations, you may request that we remove your personal registration details at any time. (Remove my information/details). Please contact the publication office if you have any questions.
