# Supplementary material for: Pre-warming before general anesthesia with isoflurane delays the onset of hypothermia in rats
Source: PLoS One. 2020 Mar 3;15(3):e0219722. doi: 10.1371/journal.pone.0219722 (PMC7053737; doi:10.1371/journal.pone.0219722)
Supplement: Attachment — Submitted filename: response to reviewers_PONE-D-19-18070R1.rtf [file pone.0219722.s002.rtf]

PONE-D-19-18070R1
Pre-warming before general anesthesia with isoflurane delays the onset of hypothermia in rats


	An unmarked version of your revised paper without tracked changes. This file should be uploaded as separate file and labeled 'Manuscript'.

Journal Requirements:
 
When submitting your revision, we need you to address these additional requirements.

1. Please ensure that your manuscript meets PLOS ONE's style requirements, including those for file naming. The PLOS ONE style templates can be found at http://www.journals.plos.org/plosone/s/file?id=wjVg/PLOSOne_formatting_sample_main_body.pdf and http://www.journals.plos.org/plosone/s/file?id=ba62/PLOSOne_formatting_sample_title_authors_affiliations.pdf
>>Done: files have been re-named as required and a few changes made to the manuscript to adhere to the journal style guidelines. Sorry for this oversight.

2. We note that you have stated that you will provide repository information for your data at acceptance. Should your manuscript be accepted for publication, we will hold it until you provide the relevant accession numbers or DOIs necessary to access your data. If you wish to make changes to your Data Availability statement, please describe these changes in your cover letter and we will update your Data Availability statement to reflect the information you provide.
>>Understood. We will make available the link to access data in the repository following acceptance.

3. Thank you for stating the following in the Financial Disclosure section:

'Natural Sciences and Engineering Research Council (NSERC) Discovery Grant (ID:
424022-2013; DSJP), Fondation Lévesque (DSJP). The funders had no role in study
design, data collection and analysis, decision to publish, or preparation of the
manuscript.'

We note that one or more of the authors are employed by a commercial company: 3Vetronic Services Ltd.

1. Please provide an amended Funding Statement declaring this commercial affiliation, as well as a statement regarding the Role of Funders in your study. If the funding organization did not play a role in the study design, data collection and analysis, decision to publish, or preparation of the manuscript and only provided financial support in the form of authors' salaries and/or research materials, please review your statements relating to the author contributions, and ensure you have specifically and accurately indicated the role(s) that these authors had in your study. You can update author roles in the Author Contributions section of the online submission form.
>>Done.

Please also include the following statement within your amended Funding Statement.

"The funder provided support in the form of salaries for authors [insert relevant initials], but did not have any additional role in the study design, data collection and analysis, decision to publish, or preparation of the manuscript. The specific roles of these authors are articulated in the 'author contributions' section."

If your commercial affiliation did play a role in your study, please state and explain this role within your updated Funding Statement.

>>Thank you for your guidance regarding the Funding Statement. It has been revised in the manuscript to reflect the role of Vetronic Services Ltd. in providing research materials (a copy of the statement is also provided in the cover letter, as requested below). We have phrased this statement as we feel appropropriate but have no objections to editorial changes (assuming the described roles of each funder are unchanged).

2. Please also provide an updated Competing Interests Statement declaring this commercial affiliation along with any other relevant declarations relating to employment, consultancy, patents, products in development, or marketed products, etc.
>>Done. Provided in cover letter.

Within your Competing Interests Statement, please confirm that this commercial affiliation does not alter your adherence to all PLOS ONE policies on sharing data and materials by including the following statement: "This does not alter our adherence to PLOS ONE policies on sharing data and materials." (as detailed online in our guide for authors http://journals.plos.org/plosone/s/competing-interests) . If this adherence statement is not accurate and there are restrictions on sharing of data and/or materials, please state these. Please note that we cannot proceed with consideration of your article until this information has been declared.
>>Done.

Please include both an updated Funding Statement and Competing Interests Statement in your cover letter. We will change the online submission form on your behalf.
>>Done, thank you.

Please know it is PLOS ONE policy for corresponding authors to declare, on behalf of all authors, all potential competing interests for the purposes of transparency. PLOS defines a competing interest as anything that interferes with, or could reasonably be perceived as interfering with, the full and objective presentation, peer review, editorial decision-making, or publication of research or non-research articles submitted to one of the journals. Competing interests can be financial or non-financial, professional, or personal. Competing interests can arise in relationship to an organization or another person. Please follow this link to our website for more details on competing interests: http://journals.plos.org/plosone/s/competing-interests
 


[Note: HTML markup is below. Please do not edit.]

Reviewers' comments:

Reviewer's Responses to Questions

Comments to the Author

1. If the authors have adequately addressed your comments raised in a previous round of review and you feel that this manuscript is now acceptable for publication, you may indicate that here to bypass the "Comments to the Author" section, enter your conflict of interest statement in the "Confidential to Editor" section, and submit your "Accept" recommendation.

Reviewer #3: (No Response)


2. Is the manuscript technically sound, and do the data support the conclusions?

The manuscript must describe a technically sound piece of scientific research with data that supports the conclusions. Experiments must have been conducted rigorously, with appropriate controls, replication, and sample sizes. The conclusions must be drawn appropriately based on the data presented. 

Reviewer #3: Yes
 
 


3. Has the statistical analysis been performed appropriately and rigorously? 

Reviewer #3: Yes
 
 
 
 


4. Have the authors made all data underlying the findings in their manuscript fully available?

The PLOS Data policy requires authors to make all data underlying the findings described in their manuscript fully available without restriction, with rare exception (please refer to the Data Availability Statement in the manuscript PDF file). The data should be provided as part of the manuscript or its supporting information, or deposited to a public repository. For example, in addition to summary statistics, the data points behind means, medians and variance measures should be available. If there are restrictions on publicly sharing data—e.g. participant privacy or use of data from a third party—those must be specified.

Reviewer #3: Yes
 
 
 
 


5. Is the manuscript presented in an intelligible fashion and written in standard English?

PLOS ONE does not copyedit accepted manuscripts, so the language in submitted articles must be clear, correct, and unambiguous. Any typographical or grammatical errors should be corrected at revision, so please note any specific errors here.

Reviewer #3: No
 
 
 
 


6. Review Comments to the Author

Please use the space provided to explain your answers to the questions above. You may also include additional comments for the author, including concerns about dual publication, research ethics, or publication ethics. (Please upload your review as an attachment if it exceeds 20,000 characters)

Reviewer #3: This is a useful study, addressing an issue that is of importance when anaesthetising both human and veterinary subjects. It is unfortunate that little emphasis is given to the important implications for research animal anaesthesia - hypothermia has the potential to influence numerous animal models, the effects may last well beyond the period of anaesthesia, and hypothermia occurs commonly when anaesthetising rodents. Although this effects is more common in mice, it is also relevant in rats, and in any event the effects noted in rats are of relevance to mice.

>>Thank you Professor Flecknell for your time and efforts in reviewing our manuscript. Our response to your suggested revision appears directly below the comment.

I have one specific comment:

Redistribution of warm blood from the core to the periphery is the primary
24 mechanism in the development of hypothermia and begins following induction of
25 anesthesia.

This is correct when the anaesthetic agent produces peripheral vasodilation, but the most widely used agent for rodents, the combination of ketamin and xylazine, produces a peripheral vasoconstriction. Perhaps it might be better to say that "when using inhalational anaesthetic agents, ….

>>Thank you for the clarification; revised as suggested. Sentence now reads, "When using inhalational anesthetic agents, redistribution of warm blood….". 

There are a small number of typographical errors that could be corrected with further proof-reading of the manuscript.

>>We have re-read and run spell checking software on the manuscript and hopefully caught everything.

>>In proof reading the manuscript we noticed that the results presented in the text (time to hypothermia) did not match the data presented in Figure 2. The text has been corrected in two places: lines 35-36 and line 270. Interpretation of the results is unchanged. Figure 2: a copy and paste error was spotted in the file submitted for review - the number of asterisks indicating statistical significance was incorrect (was ****, should have been **). The p value reported in the text was correct and remains unchanged. This has been corrected and a replacement file uploaded. Interpretation of the results is unchanged.


7. PLOS authors have the option to publish the peer review history of their article (what does this mean?). If published, this will include your full peer review and any attached files.


If you choose "no", your identity will remain anonymous but your review may still be made public.


Do you want your identity to be public for this peer review? For information about this choice, including consent withdrawal, please see our Privacy Policy.

Reviewer #3: Yes: Paul Flecknell


[NOTE: If reviewer comments were submitted as an attachment file, they will be attached to this email and accessible via the submission site. Please log into your account, locate the manuscript record, and check for the action link "View Attachments". If this link does not appear, there are no attachment files to be viewed.]


While revising your submission, please upload your figure files to the Preflight Analysis and Conversion Engine (PACE) digital diagnostic tool, https://pacev2.apexcovantage.com/. PACE helps ensure that figures meet PLOS requirements. To use PACE, you must first register as a user. Registration is free. Then, login and navigate to the UPLOAD tab, where you will find detailed instructions on how to use the tool. If you encounter any issues or have any questions when using PACE, please email us at figures@plos.org. Please note that Supporting Information files do not need this step.
 
 
 


In compliance with data protection regulations, you may request that we remove your personal registration details at any time. (Remove my information/details). Please contact the publication office if you have any questions.
